# Bacillus Calmette-Guérin (BCG)-Induced Pneumonitis: A Case Report

**DOI:** 10.7759/cureus.67049

**Published:** 2024-08-16

**Authors:** Ahmed Farrag, Jhiamluka Solano, Vijaykumar Singh

**Affiliations:** 1 Acute Medicine, Scunthorpe General Hospital - Northern Lincolnshire and Goole NHS Foundation Trust, Scunthorpe, GBR; 2 Cardiology, Scunthorpe General Hospital - Northern Lincolnshire and Goole NHS Foundation Trust, Scunthorpe, GBR; 3 Education, Academy of Medical Educators, Cardiff, GBR

**Keywords:** bacillus calmette-guérin, granulomatous hepatitis, mycobacterium bovis, pneumonitis, transitional cell carcinoma

## Abstract

Bladder cancer is the second most common genitourinary (GU) malignancy worldwide. Treatment involves early cystectomy and intravesical Bacillus Calmette-Guérin (BCG), which is effective for T1 high-grade tumors and carcinoma in situ (CIS) but can cause significant side effects, including chemical and bacterial cystitis, hematuria, incontinence, pneumonitis, malaise, fever, and sepsis. We present the case of a 47-year-old male with transitional cell carcinoma (TCC, G3 pTa) treated with transurethral resection of bladder tumor (TURBT) who developed a fever and non-productive cough after BCG injections. Initially discharged, he returned with worsened symptoms. His vital signs showed a fever of 38.2°C, a heart rate of 104 beats per minute (bpm), and a saturation of 93% on room air. Blood tests indicated inflammation and liver dysfunction. Imaging revealed lung micronodularity, and further CT imaging showed bilateral miliary nodules indicative of BCG pneumonitis. MRI ruled out disseminated tuberculosis, identifying a hepatic cyst. Cultures from blood, urine, sputum, and broncho-alveolar lavage were negative, but granulomatous inflammation was confirmed on liver biopsy. The patient was treated with oral glucocorticoids and anti-tuberculosis medications (rifampicin, isoniazid, and ethambutol), and clinical improvement was shown. The patient was discharged, and a follow-up at the respiratory clinic was scheduled. BCG pneumonitis, a severe BCG therapy complication, necessitates early diagnosis and management to reduce morbidity and mortality.

## Introduction

Genitourinary (GU) malignancies represent 24% of new cancer diagnoses, with bladder cancer being the second most common, and represent a five-year survival of 77.9% when confined to the bladder but reduced to 8.3% when metastatic [1]. Management is tailored to an early cystectomy with adjuvant treatment with intravesical Bacillus Calmette-Guérin (BCG) [2]. BCG immunotherapy post-transurethral resection of bladder tumor (TURBT) has historically been shown to reduce the recurrence of superficial cancer [3]. The immunotherapeutic effect of BCG in bladder cancer has been studied and described over the last 50 years. Animal models have shown that BCG induces an increased cluster of differentiation 4 (CD4) T-cells infiltrating the tumor. CD4 T-cells produce interferon gamma (IFN-γ), a cytokine essential in tissue homeostasis, immune and inflammatory responses, and tumor immunosurveillance [4].

Treatment with BCG has proven to be efficient in T1 high-grade tumors and carcinoma in situ (CIS). However, moderate to severe side effects have been described [5]. Local side effects include chemical cystitis, bacterial cystitis, urinary frequency, macroscopic hematuria, and urinary incontinence. Systemic side effects are general malaise, fever, and a slight occurrence of sepsis [6]. Other side effects have been described in the literature. We present a case report of a patient who developed pneumonitis in the context of intravesical BCG immunotherapy for bladder cancer.

## Case presentation

**Figure 1:**
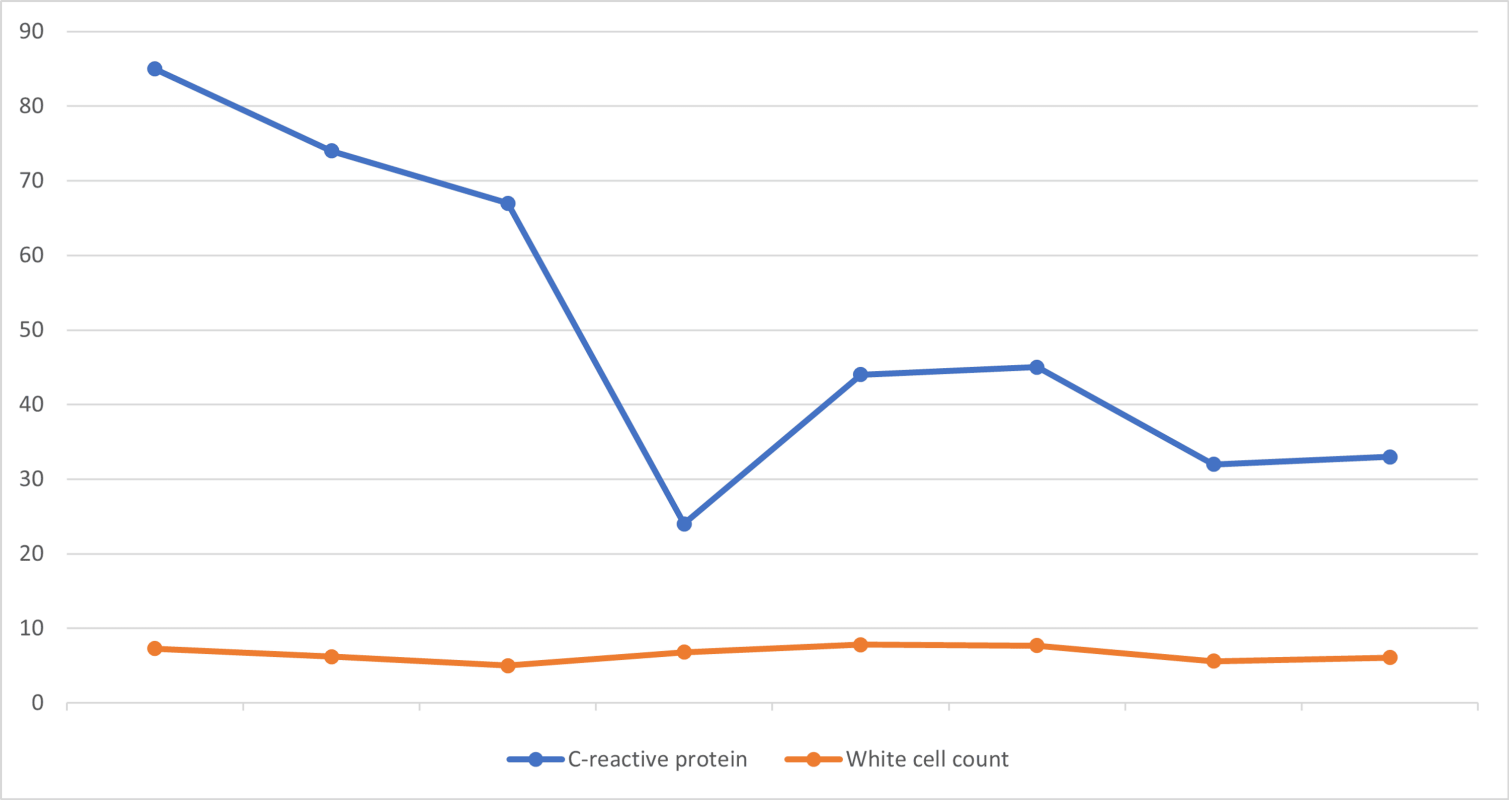
C-reactive protein changes against white cell count.

**Figure 2:**
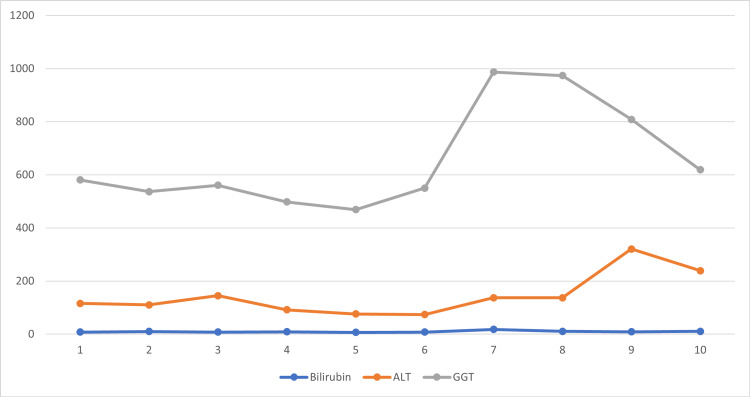
Changes in liver function tests. ALT, alanine transaminase; GGT, gamma-glutamyl transferase

**Figure 3:**
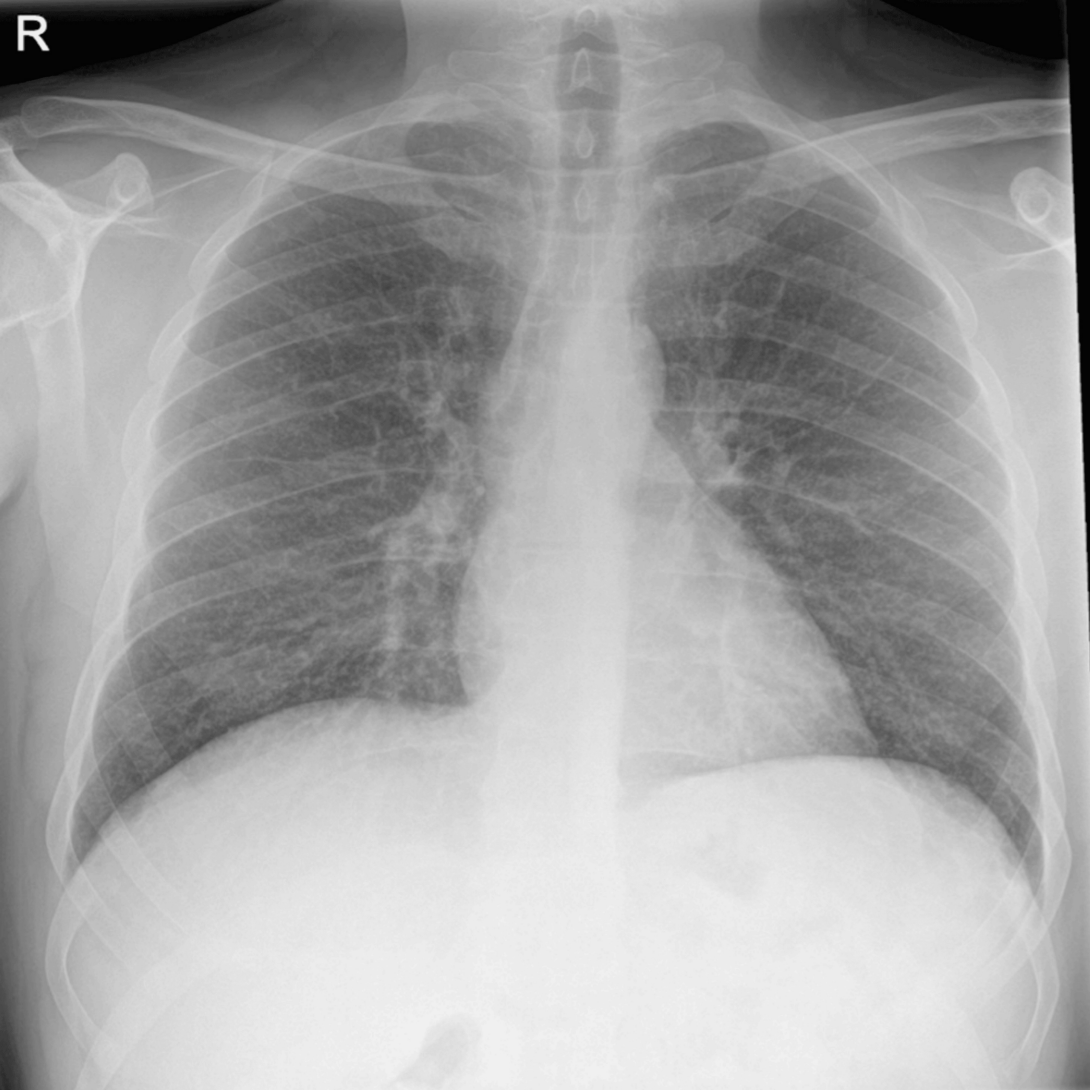
Chest X-ray on admission. PA projection shows normal heart size, no active lung lesion, clear pleural cavities, and hilar or mediastinal abnormality. PA: posteroanterior

A 47-year-old male patient with a known diagnosis of transitional cell carcinoma (TCC, G3 pTa) of the urinary bladder managed with transurethral resection of bladder tumor (TURBT) presented to the hospital to continue with ambulatory management with intravesical BCG injections. The patient developed a fever and associated non-productive cough during the procedure after the second and third injections. After close observation, the patient was considered clinically fit for discharge. A week after being discharged, the patient presented to the emergency department with a worsening non-productive cough, fever, rigors, and a generally unwell feeling. Physical examination was unremarkable with the following observations: temperature of 38.2°C, heart rate of 104 beats per minute (bpm), respiratory rate of 17 respirations per minute (rpm), blood pressure of 132/77 mmHg, and a saturation of 93% on room air. Initial investigations reported a raised C-reactive protein (CRP), a normal white cell count, and a deranged liver function test (see Figures 1, 2). A chest X-ray on admission revealed no acute changes (see Figure 3). Additional imaging investigations (CT of the abdomen and pelvis) were completed to rule out any intra-abdominal pathology, but this was reported as normal. However, micronodularity within the lung bases was noted, suggesting an ongoing inflammatory/infectious process.

**Figure 4:**
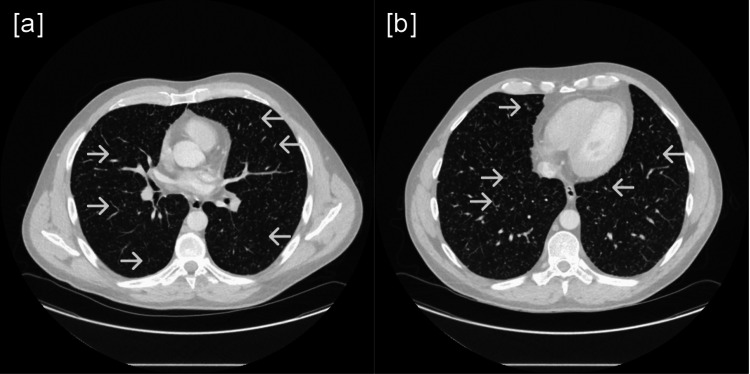
Bilateral miliary non-cavitating nodules with clear pleural spaces and no adenopathy (a and b). Examples are marked with arrows.

Following admission, we performed a CT of the thorax with contrast, revealing bilateral miliary non-cavitating nodules throughout suggestive of BCG pneumonitis (see Figure 4). An MRI of the liver was performed to rule out disseminated tuberculosis and hepatic caseating lesions. The MRI showed a subcapsular hepatic cyst noted posteriorly in segment VII, which was later biopsied with ultrasound guidance testing negative for acid alcohol fast bacteria (AAFB), Gram staining, white blood cells, extended wound culture, HIV, and QuantiFERON-TB Gold test. However, the biopsy confirmed granulomatous hepatitis. Additionally, the patient had three sets of blood cultures, a urine culture, a sputum culture for acid-fast bacilli, and a broncho-alveolar lavage, which all resulted in negative. Considering the results of the investigations, the diagnosis of BCG pneumonitis was confirmed. During his admission, the patient was started on oral glucocorticoid, which was later stopped once the diagnosis was confirmed and managed, followed by anti-tuberculosis medications (rifampicin, isoniazid, and ethambutol). The patient was discharged once inflammatory markers and clinical state improved, and a follow-up was arranged as an outpatient in the respiratory clinic.

## Discussion

BCG is a live-attenuated strain of *Mycobacterium bovis*, which is used as a vaccine against tuberculosis and has been widely used as an adjuvant immunotherapy for patients who had a transurethral resection of bladder tumor. While generally well-tolerated, some studies suggest that 95% of people have no side effects [7]; some recorded complications are self-limiting, such as a low-grade fever and malaise that should subside within 48 hours and do not require any treatment. However, some may require medical attention, such as granulomatous prostatitis (0.9%) or epididymitis (0.4%), contracted bladder (<0.3%), systemic infections such as granulomatous nephritis and abscesses (<0.3%), and pneumonitis and hepatitis (0.7%) [8]. In this report, we discuss a case of BCG pneumonitis, which is one of the most severe and life-threatening complications with an incidence of 0.7%. In 2020, 16 cases of BCG pneumonitis had been reported, including three mortalities [9]. Clinical presentation often includes nonspecific constitutional symptoms such as fever, rigors, and malaise; other cases present with respiratory symptoms varying from mild to severe cough and shortness of breath. Our case presented with persistent fever and a non-productive cough associated with shortness of breath. BCG pneumonitis should be considered one of the differentials in patients presenting with such symptoms and undergoing BCG treatment.

A detailed medical history, clinical examination, and routine investigations, e.g., full blood count, C-reactive protein levels, liver function test, and plain chest X-rays, can help tailor further investigations. Specific investigations can be aimed to confirm the diagnosis and exclude differentials with AAFB sputum cultures, urine tests for legionella, and CT of the thorax. Furthermore, invasive procedures include broncho-alveolar lavage [9]. Our case presented an additional clinical concern due to the deranged liver function tests, which we initially thought to be caused by either a caseating liver lesion or granulomatous hepatitis, a rare complication of the hematogenous spread of BCG. The first was ruled out by an MRI of the liver, and the second was confirmed by biopsy. Management usually involves oral glucocorticoid therapy and anti-tuberculosis medications [10]. Initially, our patient was started on prednisolone 40 mg and then was commenced on anti-tuberculous treatment (ATT) with isoniazid, rifampicin, and ethambutol.

BCG pulmonary complications can have devastating outcomes and are known to have a high mortality. Complications include non-caseating granulomas of the lungs, miliary tuberculosis, lymphocytic pleurisy, and severe respiratory failure. Management is primarily aimed at reducing the inflammatory process and providing tuberculosis prophylaxis using ATT; however, when complications such as hypoxemic respiratory failure arise, noninvasive or invasive mechanical ventilation can be considered [11,12].

## Conclusions

BCG immunotherapy can cause significant side effects such as pleurisy, granulomatous prostatitis or epididymitis, contracted bladder, granulomatous nephritis and abscesses, pneumonitis, hepatitis, and osteomyelitis. BCG pneumonitis is an uncommon occurrence and is considered one of the most severe complications caused by BCG immunotherapy. Early recognition and management are critical to prevent further morbidity and mortality. Providing patients with adequate education regarding side effects and red flags is essential to aid early recognition and medical intervention. In our case, the patient had a good outcome due to the early recognition of the symptoms and an appropriate management plan. Additionally, further research is required to establish clear guidelines for managing such cases.
